# Pneumatosis intestinalis in a radioactive iodine-refractory metastasic thyroid papillary carcinoma with BRAF^V600E^ mutation treated with dabrafenib–trametinib: a case report

**DOI:** 10.1186/s13256-020-02581-9

**Published:** 2021-03-02

**Authors:** M. C. Martín-Soberón, S. Ruiz, G. De Velasco, R. Yarza, A. Carretero, D. Castellano, J. M. Sepúlveda-Sánchez

**Affiliations:** 1 Medical Oncology Department, University Hospital 12 de Octubre, Madrid, Spain; 2 Nuclear Medicine Department, University Hospital 12 de Octubre, Madrid, Spain

**Keywords:** Case report, Pneumatosis intestinalis (PI), Targeted therapies, Dabrafenib, Trametinib, Thyroid cancer

## Abstract

**Background:**

Pneumatosis intestinalis (PI) is a rare entity which refers to the presence of gas within the wall of the small bowel or colon which is a radiographic sign. The etiology and clinical presentation are variable. Patients with PI may present either with chronic mild non-specific symptoms or with acute abdominal pain with peritonitis. Some cases of intestinal pneumatosis have been reported as adverse events of new oncological treatments such as targeted therapies that are widely used in multiple tumors.

**Case presentation:**

A 59-year-old caucasian female with radioactive iodine-refractory metastatic thyroid papillary carcinoma with *BRAF*^V600E^ mutation was treated with dabrafenib and trametinib as a compassionate use. After 4 months treatment, positron emission tomography–computed tomography (PET–CT) showed PI. At the time of diagnosis, the patient was asymptomatic without signs of peritonitis. The initial treatment was conservative and no specific treatment for PI was needed. Unfortunately, after dabrafenib–trametinib withdrawal, the patient developed tumor progression with significant clinical worsening.

**Conclusions:**

This case report is, in our knowledge, the first description of PI in a patient treated with dabrafenib–trametinib. Conservative treatment is feasible if there are no abdominal symptoms.

## Background

Pneumatosis intestinalis is a rare condition characterized by the presence of subserosal and submucosal gas, with air-filled cysts occurring anywhere in the gastrointestinal tract [1]. PI often presents as an incidental finding on abdominal imaging in asymptomatic patients, but it may occur in the context of life-threatening intestinal pathology, such as acute intestinal ischemia [2]. The pathogenesis is poorly understood and PI is associated with a wide range of etiologies. Chemotherapy has been defined as a well-known predisposing factor by oncologists [3]. Nowadays, due to the increased use of targeted therapies, PI has also been described as a side effect of multiple targeted anticancer drugs [4]. This entity is one of the few conditions where a pneumoperitoneum has no mandatory indication for laparotomy [5]. Although the association of tyrosine kinase inhibitors with PI is rare, its knowledge and management are essential in the era of these targeted therapies.

## Case presentation

A 59-year-old caucasian female was diagnosed with thyroid papillary carcinoma after total thyroidectomy in 2001. Diagnosed with postsurgical hypothyroidism under treatment with levothyroxine, 100 micrograms per day. There was no other previous medical history of interest. The patient did not consume tobacco or alcohol.

**Fig. 1:**
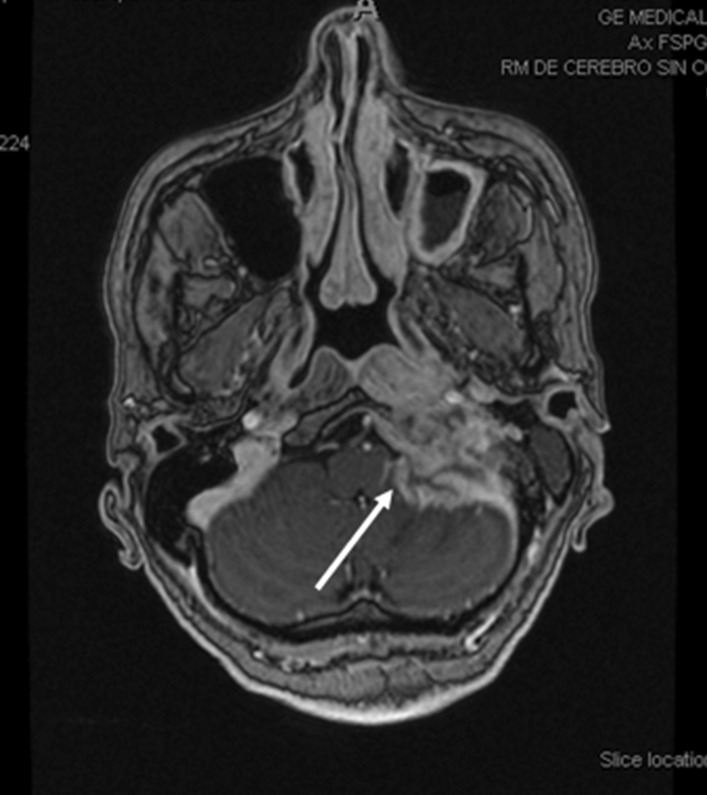
Magnetic resonance imaging June 2018 showing metastatic lesion in the skull base with destruction of bony structures

In 2008, a computerized tomography scan (CT) showed locoregional relapse and surgery was performed with resection of locoregional recurrence and left cervical lymphadenectomy. In November 2011, pulmonary relapse was treated with I-131 since November 2011 until March 2012 (total accumulated dose: 850 mCi). In October 2016, a CT scan showed a progression of the disease with cervical and pulmonary progression. The patient started sorafenib, 400 mg twice a day. Stable disease was maintained during 20 months. In June 2018, patient presented an episode of abrupt instability and cervical pain. The magnetic resonance imaging (MRI) (Fig. 1) showed a new metastatic lesion in the skull base with destruction of bony structures of the left occipital-petrous region. At this point, a molecular study of the cervical node was performed and a mutation in *BRAF*^*V600E*^ was found.

**Fig. 2:**
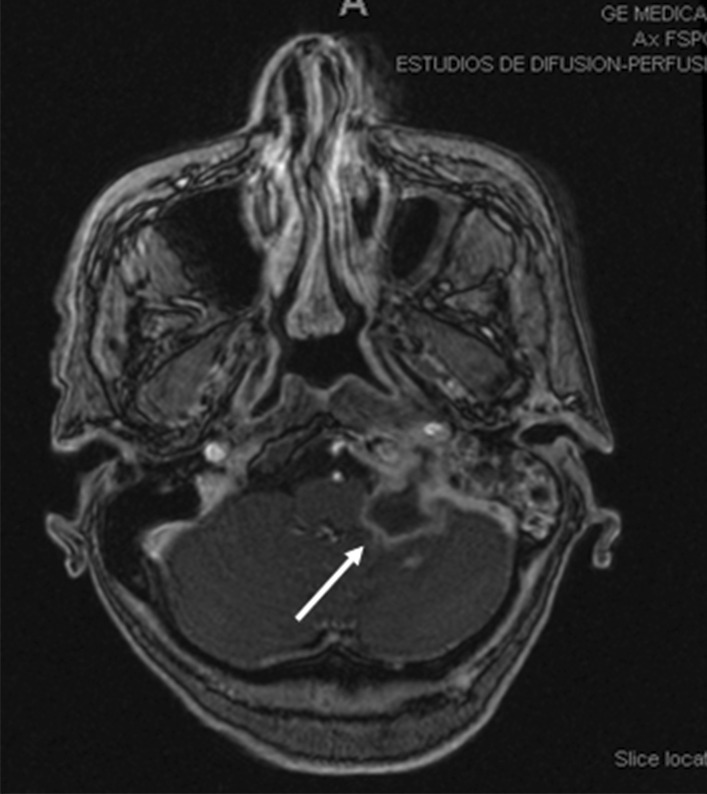
Magnetic resonance imaging October 2018 showing a slight decrease of the metastatic lesion at the base of the skull

Due to the lack of alternative therapeutic options, treatment with vemurafenib–trametinib was requested as a compassionate use. In August 2018, patient was started on the combination of dabrafenib 150 mg twice a day and trametinib 2 mg once a day. MRI in October 2018 showed a slight decrease of the metastatic lesion in the skull base (Fig. 2). In addition, the patient showed evident clinical improvement with decreased initial headache and cervicalgia.

**Fig. 3:**
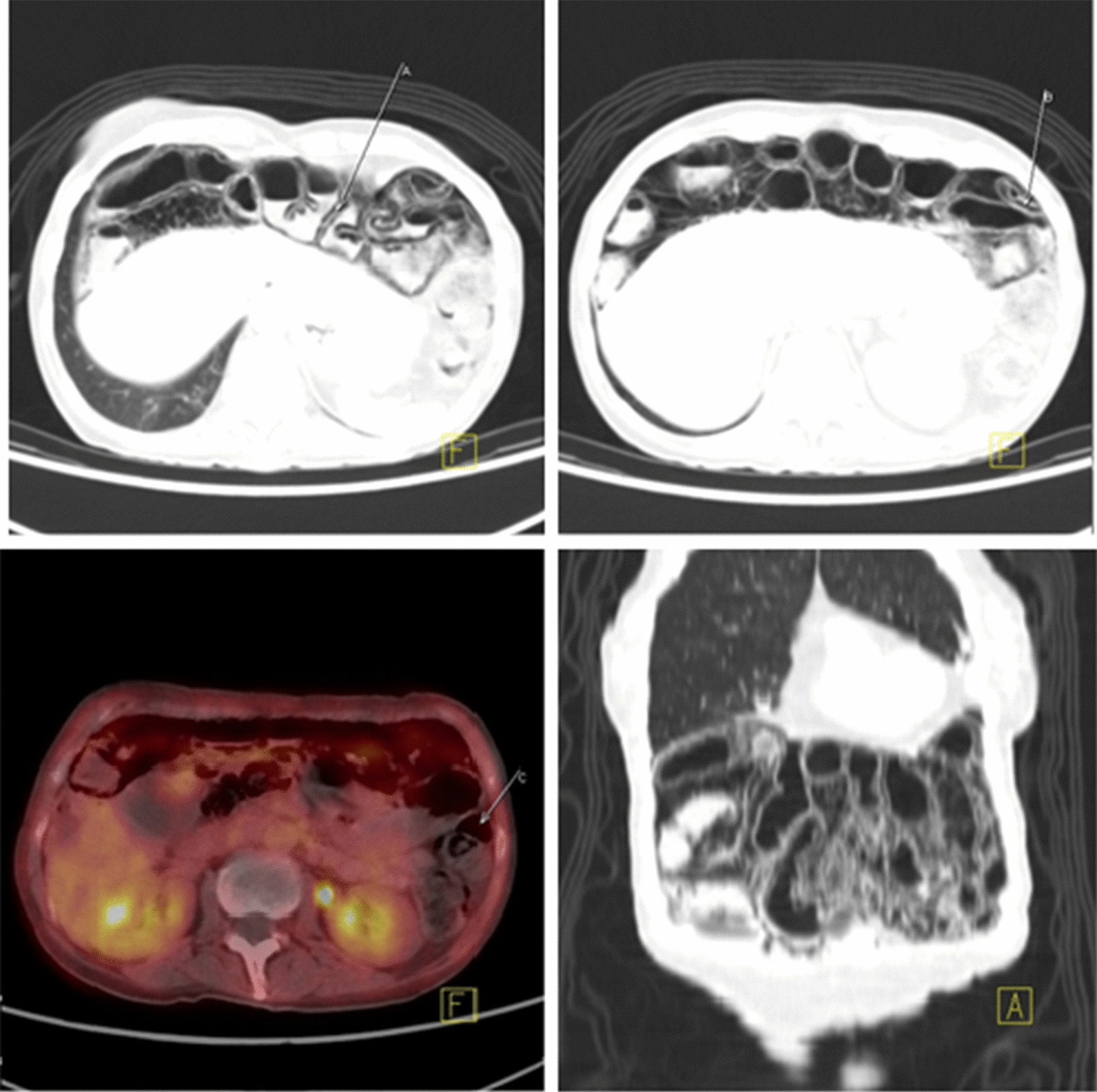
Positron emission tomography–computed tomography January 2019. (Images **a** and **b** show radiological signs of PI with mild sign of pneumoperitoneum showed in image **c**.)

A follow-up PET–CT scan was performed in January 2019. Tumor was on radiological partial response. In addition, there was intestinal pneumatosis with mild sign of pneumoperitoneum (Fig. 3). Patient had no digestive symptoms and the abdominal medical examination was completely normal. Also normal neurological examination was verified. Routine physical examination showed blood pressure 110/60 mmHg, heart rate 80 bpm and 36.5 degree centigrade temperature. Blood test showed normal liver function: AST 21 U/L, ALT 16 U/L, bilirubin 0.19 mg/dL and normal renal function: creatinine 0.7 and glomerular filtrate > 90 mL/min. Blood count values were normal: leukocytes 7.6 × 1000/µL, hemoglobin 12 g/dL and platelets 417 × 1000/µL.

The surgery department recommended conservative treatment unless new abdominal signs or symptoms were seen. Intravenous metoclopramide 10 mg/8 h and paracetamol 1000 mg/8 h were administrated. Both drugs, dabrafenib and trametinib, were discontinued after the PI diagnosis.

Only 10 days after the discontinuation of targeted therapy, tumor progression was shown with clinical deterioration due to intracranial hypertension and the patient died 4 weeks later because of intracranial disease progression. Because the cause of death was related with tumor progression, autopsy was not performed.

## Discussion

Despite PI being related to targeted therapies, we have not found any report in patients receiving dabrafenib–trametinib. Here, we presented a case of a 59-year-old woman who developed PI 5 months after starting the combination treatment with those drugs.

Papillary thyroid cancer is the most common type among all thyroid tumors. Outcome of refractory radioactive iodine tumors is poor, the 10-year survival is 10% from the time of detection of metastasis [6]. About half of papillary thyroid cancers harbor the *BRAF*^*V600E*^ mutation. Although the value of this mutation is still under investigation, thyroid cancer harboring *BRAF*^*V600E*^ mutation have worse prognosis [7]. In the last decade, tyrosine kinase inhibitors have been approved and used for radioactive iodine-refractory patients.

Vemurafenib and dabrafenib potently inhibit *BRAF* proteins containing the *V*^*600E*^ mutation and are indicated for patients with non-resectable or metastatic melanoma associated with this mutation [8]. Both drugs act in the *RAS–RAF–MEK–ERK* pathway which is overactivated by oncogenic mutation in the *BRAF* protein, triggering overactivation of this pathway and increasing cell proliferation, cell survival and angiogenesis [9]. Dabrafenib inhibits mutated *BRAF* proteins and trametinib inhibits *MEK*. Neither dabrafenib nor trametinib is currently approved for papillary thyroid cancer, however responses have been observed to those drugs in thyroid tumors carrying the *BRAF*^*V600*^ mutation.

In the last decade, the extended use of those targeted therapies has caused new types of toxicities. PI is a multifactorial entity with a wide range of etiologies: changes of the intestinal wall, peritonitis, bowel distention and corticosteroid therapy are some of the described etiologies [10].

A wide study evaluating the association of targeted therapies with PI and bowel perforation was published by Shinagare AB et al. These authors retrospectively reviewed 48 patients with cancer who developed one of these abdominal complications. Twenty-four patients were receiving molecular targeted therapies and have no other risk factors for PI or bowel perforation. Investigators showed that bevacizumab (*n* = 14) and sunitinib (*n* = 6) were the most common drugs associated with PI. Other drugs included were sorafenib, cetuximab, erlotinib and ipilimumab [11]. In the context of those treatments, the precise mechanism that leads to the association between targeted therapies and PI is currently unknown. In the case of antiangiogenics, bevacizumab, an *anti-VEGF* monoclonal antibody, has been shown to compromise the bowel wall integrity, producing intestinal wall disruption due to necrosis of the serosa and PI [11].

## Conclusion

Despite the existence of other targeted therapies associated with PI, to our knowledge, this is the first report of PI in a patient receiving dabrafenib–trametinib. Conservative treatment is feasible if there are no abdominal signs or symptoms. However, the discontinuation of the cancer treatment led to a clinical deterioration and progression of the thyroid cancer. Understanding the toxicity of novel treatments is crucial in the management of our patients. In patients who are receiving targeted therapies it is possible that PI, if it appears, determines the vital prognosis and it should be considered a severe adverse event.

## Abbreviations

PI: Pneumatosis intestinalis
PET–CT: Positron emission tomography–computed tomography
CT: Computed tomography
MRI: Magnetic resonance imaging
VEGF: Vascular endothelial growth factor

## Availability of data and materials

This case report corresponds to a real case diagnosed and treated in our clinical center. Clinical and radiological data presented correspond to real data. The datasets supporting this article are stored in Hospital 12 Octubre medical records.
